# Longitudinal Analysis of the Impacts of Urogenital Schistosomiasis on the Gut microbiota of Adolescents in Nigeria

**DOI:** 10.21203/rs.3.rs-2832346/v1

**Published:** 2023-04-27

**Authors:** Olumide Ajibola, Swathi Penumutchu, Bashar Gulumbe, Uzairu Aminu, Peter Belenky

**Affiliations:** First Technical University; Brown University; Federal University Birnin Kebbi; Federal University Birnin Kebbi; Brown University

## Abstract

The gut microbiome is important for many host physiological processes and helminths and these interactions may lead to microbial changes. We carried out a longitudinal study of the impacts of S. haematobium infection on the gut microbiome of adolescents (11–15 years) in northern Nigeria pre and post praziquantel treatment. Using 16S sequencing a total of 267 DNA from faecal samples of infected versus uninfected adolescents were amplified and sequenced on an Illumina Miseq. We assessed the diversity of the taxa using alpha diversity metrices and observed that using Shannon index we obtained significant differences when we compared infected samples at 3, 9 and 12 months to baseline uninfected controls (P= <0.0001, P=0.0342 and P=0.0003 respectively). Microbial community composition analysis revealed that there were only significant differences at 3, 9 and 12 months (P=0.001, P=0.001, P=0.001 and P=0.001, respectively). We also demonstrated that the effects of the infection on the gut was more significant than praziquantel. Overall, our data suggests that S. haematobium, a non-gut resident parasite has indirect interactions with the gut. The bacterial taxa changes we have identified opens up the opportunity to investigate their role in human health, especially in urogenital schistosomiasis endemic communities.

## Introduction

Schistosomiasis, also known as bilharzia, is prevalent in children due to lack of good hygiene practices, access to clean water, and poor health care facilities in affected countries such as Nigeria^1^. There are at least 240 million people infected annually worldwide, with 93% of those infected located in sub-Saharan Africa (SSA)^2,3^. Schistosomes have a complex life cycle involving humans, snails, and freshwater. There are three main species of *Schistosoma* that cause human schistosomiasis, *S. mansoni*, *S. haematobium* and *S. japonicum*^4^. In SSA, *S. haematobium* is the main causative agent of urogenital schistosomiasis^5,6^. Adult male and female schistosomes can be found in the veins of their human hosts, and schistosomula, a specific life cycle stage of the parasite can migrate through lymphatics and blood circulatory systems to the lungs and liver^7^. Ultimately, parasites reside within the perivascular or mesenteric venules. Fertilised eggs are shed in faeces or urine through the mating of adult male and female worms that arrive at the mesenteric veins of the lower plexus of the large intestine. Diagnosis of schistosomiasis is primarily through microscopic examinations of urine or faeces. Treatment is administered using praziquantel, and endemic countries engage in regular rounds of mass drug administration campaigns^7,5,8^.

The gut microbiome is important for many host physiological processes, including the development of the immune system and other systemic changes^9,10^. The microbiome of humans has been described to be associated with many communicable and non-communicable diseases. Helminths, have the ability to colonise the same environment as bacteria or have indirect systemic impacts on the gut and these interactions may lead to microbial changes in the gut^11,12^. Helminths have also been demonstrated to skew immune responses towards a T-helper 2 cell response and modulate the gut microbiome promoting the bloom in bacteria species such as *Prevotella* and *Proteobacteia* species^13–15^. *Proteobacteria* is seen as a marker of dysbiosis in the intestinal microbiome, as well as a critical determinant of host’s health, metabolism and inflammation^16^. Increases in *Proteobacteria* largely attributed to a bloom in *Enterobacterales* is associated with oxygen which contributes to microbiome and systemic inflammation which could affect infection prognosis^17–21^. In a recent study in Zimbabwe, Kay *et al.*, reported differences in the microbiome structure of children infected with *S. haematobium* versus uninfected, with no reversal of the microbiome structure post praziquantel treatment. They also showed an abundance of *Proteobacteria* species in their infected cohorts^14^. Studies in Australia and Ecuador have also shown that helminth infections increased overall bacterial diversity in infected versus uninfected cohorts^22,23^.

In our previous work, we profiled the faecal microbiome of adolescents (ages 11 to 15 years) positive for *S. haematobium* versus uninfected controls. We demonstrated that the microbiomes of infected children differed dramatically from the uninfected group. Specifically, *S. haematobium*-positive subjects had more diverse microbiomes as measured by observed operational taxonomic units (OTUs). There was also a reduction in Firmicutes and an elevation in *Proteobacteria* which is a classic indication of microbiome dysbiosis. While this work is a strong indicator that *S. haematobium* is associated with microbiome differences it was lacking a longitudinal perspective or the impact of praziquantel therapy which may be required to establish the parasite as the cause of gut microbiome changes. There are limited studies from Africa that have examined the association between helminth infections and gut microbiome^13^. To understand the interaction of *S. haematobium* and the gut microbial community, we carried out a one-year longitudinal analysis of 267 stool samples of *S. haematobium* infection on the gut microbiome of adolescents (11–15 years) pre and post praziquantel treatment in Kebbi State, northwest Nigeria where urogenital schistosomiasis is endemic. We accounted for factors such as age, sex, diet, disease states, and other conditions that could influence the gut microbiome. Here, we describe the impacts of *S. haematobium* interactions with the gut microbiome on a longitudinal basis with samples collected at five time points.

## Materials and Methods

### Ethics Statement

This study was approved by the Kebbi State Ministry of Health, Nigeria registration number KSHREC 106:47/2021. All research was undertaken in accordance with the relevant guidelines and regulations of the Kebbi State Ministry of Health. The study and its risks were explained to the participants, who then verbally assented to participation if they were interested. Parents and or legal guardians of adolescents were requested to provide informed consent for their child’s participation by signing the study consent form; in the case of illiteracy, thumbprinting was used, as approved by the State Ministry of Health Ethics Committee.

To reduce the risk of co-infection with gastrointestinal helminths, any potential subjects who reported recent gastrointestinal distress were excluded. Prior to sample collection, the parents/guardians of adolescents who had consented to their children’s participation were informed of the study as well as any associated risks.

### Study area

#### Sample collection

Stool and mid-stream urine samples were collected between 10 am and 2 pm in standard specimen collection bottles, labelled and transported to the laboratory for processing. The sedimentation technique was applied for examination of *S*. *haematobium* eggs in the urine. A minimum of 10 mL of urine was collected per participant at baseline and every three months. Urine was spun down at 1,000 × *g* for 5 minutes, the supernatant was decanted, and the sediments were examined under the 40x objective of a brightfield microscope (Olympus, USA) to identify *S*. *haematobium* eggs^13^. Stool samples were also collected from each child who provided a urine sample and stored in sterile bottles. All samples were frozen at − 20 °C within 3 hours of collection until DNA extraction. The presence of eggs in the urine was used to identify cases of adolescents with urogenital schistosomiasis. After sample collection, all the children were treated with 40 mg/kg praziquantel by the State government nurses as part of the bi-annual national schistosomiasis control program.

#### DNA extraction

Microbial DNA was extracted from a total of 267 stool samples of adolescents: 46 urogenital schistosomiasis-infected adolescents supplied stool samples at baseline, they were treated with praziquantel and followed for one year and supplied stool samples every three months (Supplementary Table 1). Fifty uninfected kids provided stool samples at baseline and only 18 of the 50 uninfected kids provided samples at six months post praziquantel treatment. All stool samples were transported to the laboratory and frozen at − 20 °C before DNA extraction. Approximately 1 g of stool was removed from the center of defrosted fecal samples and was processed following the manufacturer’s protocol using the Quick DNA Fecal/Soil Microbe MiniPrep ™ Kit ZR D6010 (Zymo Research), which utilizes robust mechanical lysis.

### 16S rRNA sequencing

#### 16S Amplicon generation

Extracted genomic DNA was quantified using Qubit2 (Invitrogen) to ensure sufficient quantity for amplification. Amplification was carried out in triplicates using the Earth Microbiome Project protocols^24^ using a library of barcoded adaptor primers and a single reverse primer (806R) to amplify the V4 hypervariable region of the 16S rRNA gene with Phusion high-fidelity polymerase. The cycling conditions used for amplicon generation were as follows: (1) initial denaturation at 98°C for 30 s, (2) 34 cycles consisting of denaturation for 10 s at 98°C, annealing at 57°C for 30 s and extension at 72°C for 30 s, followed by a (3) final extension at 72°C for 5 min. Approximately 240 ng of each amplicon was pooled together for library generation.

#### Library generation

16S amplicons were cleaned using the NucleoSpin PCR Cleanup Kit (Machery-Nagel, Düren, Germany) before submission to the Rhode Island Genomics and Sequencing Center at the University of Rhode Island (Kingston, RI, USA). Samples were pair-end sequenced (2 × 250 bp) on the Illumina MiSeq platform using the 600-cycle kit with standard protocols.

#### Data processing

##### Analysis of 16S rRNA Sequencing Reads

Raw 16S rRNA reads were subjected to quality filtering, trimming, de-noising with DADA2 (via q2-dada2)^25^. Demultiplexed reads were imported into the software package Quantitative Insights Into Microbial Ecology 2 (QIIME2 version 2021.8)^26^. Within QIIME2, raw reads were quality-filtered, denoised and merged using the Divisive Amplicon Denoising Algorithm 2 (DADA2) pipeline^25^. Ribosomal sequence variants were aligned with mafft (via q2-alignment)^27^, and phylogenetic tree construction was done with fasttree2(via q2-phylogeny)^28^. Taxonomic assignment was conducted using the pre-trained Naive Bayes classifier and the q2-feature-classifier^29^ trained on the SILVA 132 99% database^30^. Taxonomy was assigned using the 99% identity SILVA (release 119) V4 classifier^30^. Amplicon sequence variants (ASVs) were generated through traditional clustering methods. The ASV table, rooted phylogenetic tree, representative sequences, and metadata from QIIME2 were then exported for further analysis in R (V3.3.1). Differential abundance testing was performed using the DESeq2 R package^31^. Microsoft Excel version 16.68 was used to rank the top 10 taxa from the ASV tables and graphs were plotted in Graphpad prism version 9.3.1.

#### Diversity Analyses

Alpha diversity (Shannon, Faith’s phylogenetic diversity) and beta diversity (Bray-Curtis dissimilarity)^32,33^ were calculated using the phyloseq package (version 1.30.0) in R (version 3.6.2)^34–36, 37,38^. Two-tailed Mann Whitney U test (Prism v9) was used to determine the significance of differences in alpha diversity between infection groups. Beta diversity (Bray-Curtis dissimilarity) was analysed using the VEGAN (V2.4-package^39^, and PERMANOVA was used to analyse the significance of differences in beta diversity using the adonis function in phyloseq(version #.#.#). Principal coordinate analysis (PCoA) was then performed on the beta diversity distance matrices to visualize any relationships between microbiome composition and infection status.

#### Differential Abundance Analyses

Differential abundance analysis between infection groups was performed using the DESeq2 (V1.14.1) package in R with agglomeration at various taxonomic levels^31^. Significance was determined by the Benjamini, Krieger and Yekutieli test to correct for False discoveries with adjusted *p*-value < 0.05^40^.

## Results

Cohort characteristics: A total of 267 stool samples were collected over one year every three months at four-time points from 99 volunteers (46 urogenital schistosomiasis positive and 50 negative) aged between 11–15 years. There were 41 (89.1%) male and 5 female (10.9%) infected children analysed in this study (Supplementary Table 1). There were no significant differences in the dietary habits of the subjects in this study. All subjects at baseline provided urine and stool samples and were treated with praziquantel irrespective of their infection status as part of an ongoing bi-annual prophylaxis campaign for schistosomiasis. We were able to collect stool and urine samples from the infected subjects at baseline, 3, 6, 9 and 12 months, respectively. Some of the infected subjects were lost to follow up and the number of subjects that provided samples at each time point is shown in supplementary Table 1. While for uninfected adolescents, we collected samples at baseline and 6 months respectively.

### Baseline composition of the microbiome

**Figure 1.**
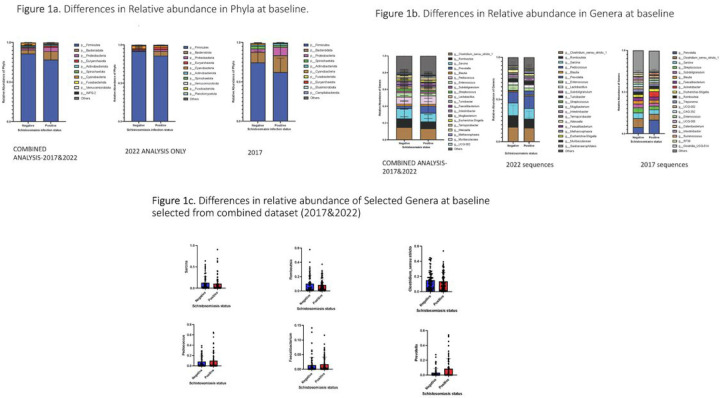
**1a.** Differences in Relative abundance in Phyla at baseline. **1b.** Differences in Relative abundance in Genera at baseline. **1c.** Differences in relative abundance of Selected Genera at baseline selected from combined dataset (2017&2022).

Previously, we have shown that there were differences in the gut microbiome of uninfected and infected kids at baseline. To ensure the robustness and reproducibility of our work, we re-analysed the sequences from 2017 using the current SILVA database^30^ and combined the sequences with sequences we generated in the current study for analysis. We began by investigating changes in community composition at higher taxonomic levels at baseline. Comparison of sequences from samples that were analysed in 2022 and re-analysed sequences from samples collected in 2017 were combined for analysis. Using excel we ranked the top 10 ASVs and found that a baseline comparison of the three groups revealed a state of dysbiosis as a result of increased *Proteobacteria, Euryarchaeota* and decrease in *Firmicutes* and *Cyanobacteria* (Fig. 1A). We also analysed the taxonomy at lower levels (Supplementary Figs. 1–2: class and order) and identified lineages that increased (*Pediococcus, Faecalibacterium* and *Prevotella*) and decreased (*Sarcina*, *Rombutsia* and *Clostridium_Sensu stricto*) (Fig. 1B-C).

### Longitudinal Changes Across Taxonomic levels

**Figure 2.**
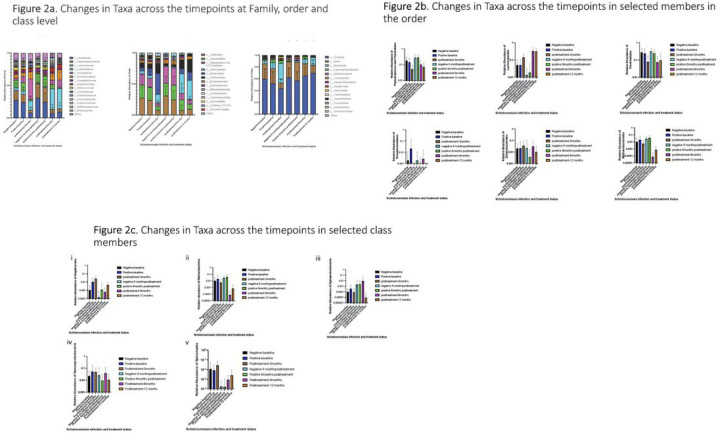
**2a.** Changes in Taxa across the timepoints at Family, order and class level **2b.** Changes in Taxa across the timepoints in selected members in the order **2c.** Changes in Taxa across the timepoints in selected class members

Once we established that there were taxonomic changes in the microbiome of infected and uninfected kids at the Phylum and Genera levels, next, we decided to investigate these changes longitudinally over one year across family, order, and class. We decided to focus our analyses using the combined 16S sequences from 2017 and 2022. At baseline, we observed an increase in *Prevotellaceae, Ruminococcaceae, Streptococcaceae, Methanobacteriaceae* and *Enterobacterales* at the family level. Family level changes at 3 months were similar to baseline, with the exception of marked increases in *Prevotellaceae* and *Ruminococcaceae* relative abundances. Family level changes at 6 months were similar to those of baseline, with the exception that *Anaerovoracaceae* was significantly reduced and *Prevotellaceae* was completely absent in both infected and uninfected subjects. At 9 and 12 months, the microbiomes were very similar, with *Anaerovoracaceae* being restored to uninfected levels at baseline (Fig. 2A). At the order level, we observed that most members were reduced in the infected compared to the uninfected group, with the exception of the presence of *Pseudomonadales*, which was absent in the uninfected kids (Fig. 2B). At 3 months, the members had a different microbiome from baseline with enrichment of *Bacterioidales, Lachnospirales* and *Oscillospirales*. While at 6 months, the microbiome of infected and uninfected subjects was similar, by later timepoints (9 and 12 months) microbiomes remained similar between infected and uninfected groups, however these microbiomes were distinct from previous timepoints (0, 3 and 6 months). There was an enrichment of *Oscillospirales, Lachnospirales* and reduced levels of *Clostridiales*. At the class taxonomic level, the main difference was observed at 3 months with a marked increase in *Bacteroidia* compared to all other time points. Additionally, *Methanobacteria* was completely absent at 3, 9 and 12 months from the microbiome of the kids (Fig. 2C).

### Differential abundance of Genera

**Figure 3.**
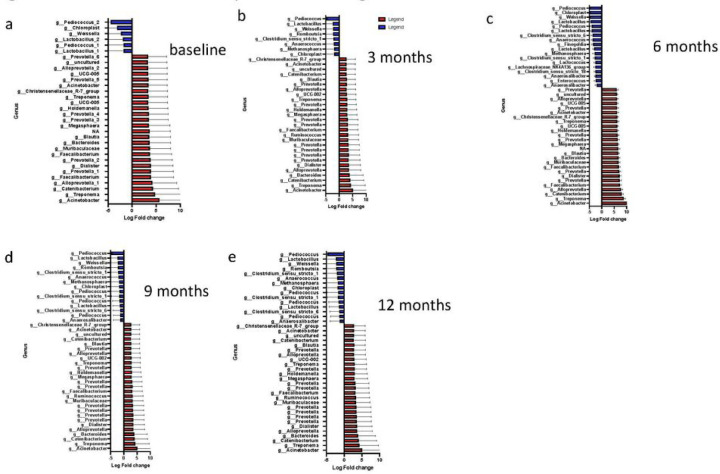
Differential expression of genera

We carried out differential abundance analysis of our samples at the genus level across all time points analysed in our study using DESeq2^31^. We identified significant differences in 32 genera at baseline. Some of the genera that increased include *Prevotella, Alloprevotella, UCG-005, Uncultured, Acinetobacter, Christensenellaceae, Treponema, Holdemanella, Megaspaera, Blautia, Bacteroides, Muribaculaceae, Faecalibacterium, Dialister* and *Catenibacterium* and those that decreased were *Lactobacillus 1 & 2, Pediococcus* 1 & 2, *Chloroplast* and *Weisella* (Fig. 3). The significant changes in genera at 3 and 6 months were similar to baseline with the addition of an increase in *Ruminococcus* at 3 months. The following decreased genera at 3 months (*Anaerococcus, Methanosphaera, Clostridium sensu stricto* and *Romboutsia*) and 6 months (*Finegoldia, Lactococcus, Lachnospiraceae NKA136, Clostridium sensu stricto, Anerosalibacter* and *Enterococcus*) were present in addition to the changes observed at baseline. The differential abundances of the bacteria present at 9 and 12 months were similar (Fig. 3) compared to those from baseline to 6 months. We observed that there was more *Prevotella* species at 9 months and 12 months, irrespective of infection status, compared to other time points in our analysis.

### Longitudinal gut microbial alpha and beta diversity analyses between Infection groups

**Figure 4.**
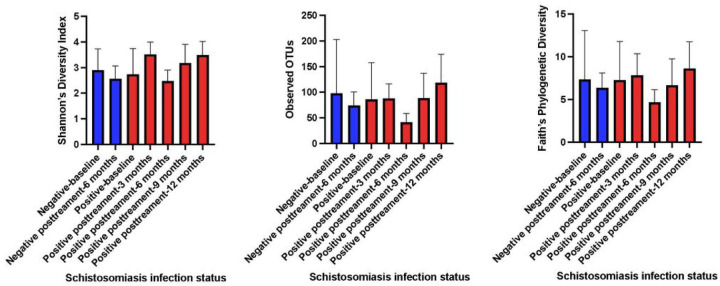
Measures of Alpha Diversity

We examined the metrics of alpha diversity to determine the diversity of taxa within each microbial community across all the time points sampled. We used two different metrics for analysis; Shannon, and Faith’s Phylogenetic Diversity (Fig. 4). We conducted a comparison of all samples that had a corresponding uninfected control at the same time point, while those that didn’t have corresponding controls were compared to baseline uninfected controls. We observed that there were no significant differences for Shannon indices at baseline and 6 months between uninfected versus infected individuals (P = 0.7847, and P = 0.2632 respectively). However, when we compared infected samples at 3, 9 and 12 months to baseline uninfected controls we observed significant differences (P = < 0.0001, P = 0.0342 and P = 0.0003 respectively). Taxonomic richness of the microbial community measured using the amplicon sequence variants (ASVs) were not significant at baseline and 9 months (P = 0.5009 and P = 0.1408), while there were significant differences at 3, 6 and 12 months (P = 0.0076, P < 0.0001 and P = 0.0023). Faith’s phylogenetic diversity was also computed and found to be significantly different at 3, 6 and 12 months (P = 0.0221, P = 0.0001 and P = 0.0088), while there were no significant differences at baseline and 9 months (P = 0.4343 and P = 0.4297). Next, we examined the beta diversity of the samples to determine the divergence in community composition between the samples collected at the various time points. This was measured using the Bray Curtis similarity index, and PERMANOVA to test for statistical differences. We observed that there were no significant differences in the microbial community composition at baseline and 6 months (P = 0.1329 and P = 0.6533). While a comparison of the baseline composition of microbial community composition to time points 3, 9 and 12 months (P = 0.001, P = 0.001, P = 0.001 and P = 0.001, respectively) were significantly different (Fig. 5).

### Effects of praziquantel on the gut microbiome

**Figure 6.**
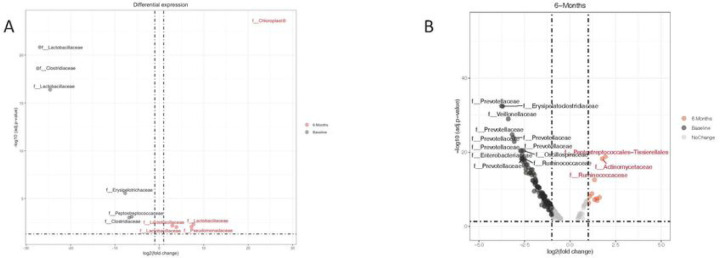
DESEq analysis for impact of praziquantel on the gut microbiome Impact of praziquantel on the gut microbiome. (A) baseline versus 6-months negative. (B) baseline vs 6-months positive showing that infected adolescents have more significant effects than praziquantel.

Following the differences observed in the microbial community composition at 3, 9 and 12 months, we asked whether these effects were as a result of the anthelminthic treatment given to the kids. To describe this, we used a DESEq analysis to determine the impact of the drug on the kids gut microbiomes across the various time points sampled. We observed that the effects of praziquantel on the gut microbiome of the subjects was minimal when compared to changes induced by the infection. In Fig. 6, it is clearly visible that the infected adolescents have more significant differences compared to praziquantel. Other analysis of other timepoints of the impacts of praziquantel revealed no significant impacts (Supplementary Fig. 3).

## Discussion

Studies on the microbiome have gained significant advances in the last decade and improved our understanding of various biological processes in the human body, identified new drug targets and treatment strategies. Here, we used 16S sequencing to investigate the longitudinal impacts of *S. haematobium* infection on the gut microbiome of adolescents in Nigeria. Several studies have described the impacts of age^41,42^, diet and the environment^43,44^ on the composition of the microbiome. Our findings are consistent with previous studies that schistosome infections have an impact on the diversity and abundance of the gut microbiome^12–15, 22,45^. In our previous work, we described signatures of dysbiosis in the gut of kids infected with urogenital schistosomiasis compared to uninfected kids at a single time point^13^. Here, we combined the sequences from our 2017 study and new longitudinal data from 2022 at five timepoints, reanalysed the data, and our analysis still revealed a gut microbiome that was reminiscent of a state of dysbiosis. The reduction in Firmicutes abundance was more in the 2017 sequences compared to the 2022 or the combined sequences from both years (2017 and 2022). *Firmicutes* have been positively associated with soil-transmitted helminth infections^46^. At the genus level, we observed baseline differences in *Clostridium_sensu_stricto_1, Romboutsia, Sarcina, Prevotella* and other genera that have been demonstrated to be important members of the gut microbiome^47^. Similar to previous studies, we also demonstrate that in addition to the reduction in Firmicutes abundance, a major difference at the Phyla level between *S. haematobium* infected and uninfected kids was an increase in *Proteobacteri*a in the infected cohorts. Some of the bacterial taxa changes we observed at baseline were similar to published reports from Zimbabwe where 16S and metagenomic analysis were done on stool samples *S. haematobium* infected kids^14,15^.

We also describe the longitudinal impacts of *S. haematobium* infection over a period of one year at five-time points. Most studies on the impacts of helminths on the gut microbiome have been single time points or collected data pre and post-anthelminthic treatments. This presented a unique opportunity to investigate the temporal changes that may occur in the gut microbiome pre and post-praziquantel administration in the study population. In addition, it provided an opportunity to investigate the role of a non-gut resident parasite as the cause of the microbiome changes in these kids. The temporal analysis of the significant genera changes that increased across all the time points (baseline, 3, 6, 9 and 12 months) identified genera such as *Acinetobacter, Treponema, Bacteroides, Alloprevotella* and *Prevotella* as some of the key taxa that changed. While the key genera that significantly decreased across all time points were *Pediococcus, Chloroplast, Lactobacillus and Weissella. Pediococcus* species e.g. *P. acidilactici* has been associated with improving recovery from gastrointestinal disorders and a decrease in clinical severity of atopic dermatitis^48,49^. *Weissella* species have been isolated from raw milk, fermented cereals and vegetables^50^. Some species, such as *W. cibaria*, have been identified to possess anticancer, anti-inflammatory, anti-fungal, anti-bacterial and immune-boosting properties^51,52^. *W. cibaria* is a lactic acid bacterium and has been associated with regulating the integrity of the intestinal epithelial barrier, through dampening inflammatory responses that reduce the synthesis of TNFa, IL-6 and Il-8^51,52^. The significant reduction in the presence of these bacteria in the *S. haematobium* infected group might point to increased inflammation in the kids. Some studies have described the association of certain bacteria taxa, such as *Dialister, Clostridium XIVa, Weissella* and *Bacteroides* with incomplete parasite clearance^53^.

The African diet is carbohydrate-rich, and some studies have described that *Prevotella* and *Candida* are associated with carbohydrate rich diets^54^. The kids in this study feed mostly on carbohydrate-rich foods such as *fura da nono*, rice and *kunun zaki* typical of northern Nigeria residents^55^. Indeed, we observed high *Prevotella* levels at baseline which increased significantly at later time points 9 and 12 months. The age group of the participants in this study was restricted to 11–15 years, this is important to note considering the variability associated with the microbiome structure of kids as they grow^42,56,57^. Our selection of this age group is based on our previous work, where we have demonstrated that there were no age-specific effects on the gut microbiome^13^. Owing to the peculiarity of our study population as previously reported, we could not control for possible sex-related bias, hence we had more males than females enrolled in this study. This is not unusual for a conservative Islamic population, where girls tend to be less willing to participate in studies of this nature. Previous studies have, however, reported that there were no sex-related bias on the impacts on helminth infections on the gut microbiome^13,53^. Within urogenital schistosomiasis endemic communities, several studies have reported that differences in infection patterns seen in gender is linked to increased water contact practices amongst males as opposed to females^58,59^. One of the limitations of this study was a loss to follow-up of some of the study subjects and the inability to collect samples from all uninfected kids at the time points that were analysed. This would have allowed more precise comparisons of the microbiomes as they change pre and post-praziquantel treatment over time. Another challenge with collecting uninfected samples from schistosome endemic communities is the likelihood of the kids having been infected in the past and being cured of the infection. However, we anticipated that the likelihood of the uninfected groups having obtained an infection within two years prior to the study was low. We make this assumption because there had been no mass drug administration campaign in the community for at least 2 years before our study, and all participants reported not to have taken any anthelminthic treatment within the same period.

One of the challenging aspects of microbiome research is inferring causation, especially in a community that is schistosome endemic, which we could not establish in this study. We observed significant differences in the microbial communities at 3, 9 and 12 months compared to baseline and 6 months. Further analysis revealed that infection seemed to have greater effect on the changes on the gut than praziquantel. The changes in the microbiome of adolescents could also due to declining efficacy of the drug or normal changes associated with adolescents as they grow. It is interesting to observe that *S. haematobium* worms mostly live in the pelvis venous plexus^60^, yet they exert indirect effects on the gut microbiome just like other intestinal helminths^22,45,47, 61–66^. The changes in the microbial composition of the gut through the activity of helminths have led to the hypothesis that there is a potential link between these microbial community changes and the modulation of inflammatory responses in autoimmune diseases.

In conclusion, the strength of our study is in its design and the opportunity provided to examine the dynamic relationship of the parasite with the host pre and post-anthelminthic treatment over a period of one year. It also allowed us to study the microbial community differences and any dietary or environmentally related changes that occurred in the subjects over time, specifically at later time points. To the best of our knowledge, this is the first study to investigate the longitudinal impacts of *S. haematobium* on the gut microbiome, these findings open up opportunities for future studies to examine the impacts of the bacteria taxa changes we have described in this study and its implication for human health.

## Supplementary Material

Supplement 1

## Figures and Tables

**Figure 5A-B.**
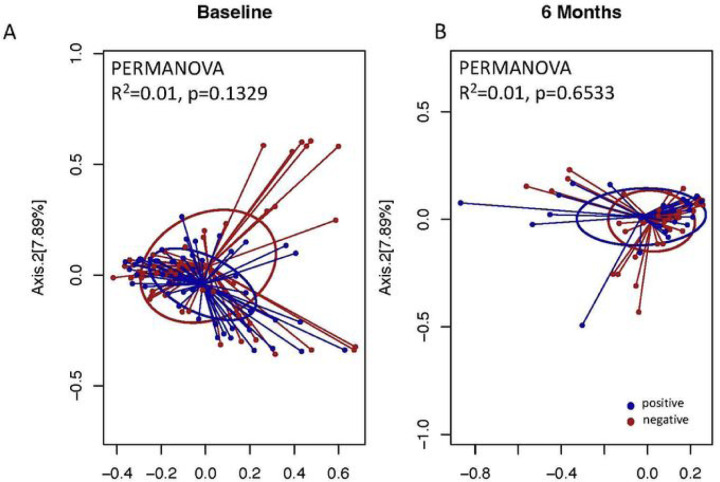
Principal coordinate analysis of community similarity by schistosomiasis infection patterns. No Significant difference between infected versus uninfected adolescents

**Figure 5C-F.**
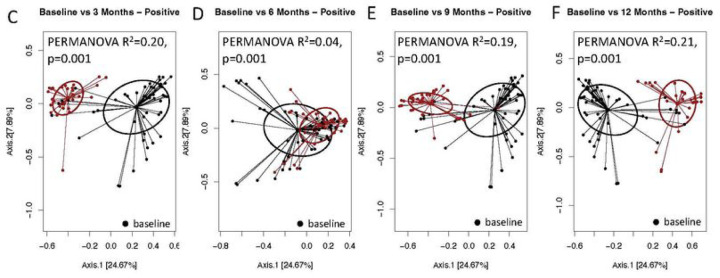
Significant differences between baseline positive patients over time
