# Supplementary material for: Longitudinal Analysis of the Impacts of Urogenital Schistosomiasis on the Gut microbiota of Adolescents in Nigeria
Source: Res Sq. 2023 Apr 27:rs.3.rs-2832346. Preprint. [Version 1] doi: 10.21203/rs.3.rs-2832346/v1 (PMC10168446; doi:10.21203/rs.3.rs-2832346/v1)
Supplement: Supplement 1 [file NIHPPRS2832346V1-supplement-1.pdf]

Supplementary Files

This is a list of supplementary files associated with this preprint. Click to download.

- SupplementaryTablepdf.pdf
- supplementaryfigures.pdf
